# High clinical burden of classical homocystinuria in the United States: a retrospective analysis

**DOI:** 10.1186/s13023-025-03530-9

**Published:** 2025-01-24

**Authors:** Mahim Jain, Mehul Shah, Kamlesh M. Thakker, Andrew Rava, Agnes Pelts Block, Colette Ndiba-Markey, Lionel Pinto

**Affiliations:** 1 Kennedy Krieger Institute, Johns Hopkins Medicine, Baltimore, MD USA; 2 Travere Therapeutics, Inc., San Diego, CA USA; 3 Notting Hill Consulting LLC, Celebration, FL USA; 4 Genesis Research Group, Hoboken, NJ USA; 5 Nemours Children’s Hospital, 1600 Rockland Road, Wilmington, DE 19803 USA

**Keywords:** Classical homocystinuria, Total homocysteine, Clinical burden

## Abstract

**Background:**

Classical homocystinuria (HCU) is a rare genetic metabolic disorder resulting in elevated homocysteine and methionine levels. The clinical characteristics and associated complications of HCU are well documented. However, there is limited published research on the clinical burden of patients with HCU, especially stratified by total homocysteine (tHcy) levels. This study aimed to describe the overall clinical burden of patients with HCU in the United States and key clinical events by tHcy levels using administrative claims data.

**Methods:**

This non-interventional retrospective cohort analysis from January 01, 2016, through September 30, 2021, used Optum’s de-identified Market Clarity Data. Patients who had 1 or more International Classification of Diseases, Tenth Revision code for homocystinuria (E72.11) or the signs, disease, and symptoms term *homocystinuria* in the natural language processing dataset were included. To obtain a study population most likely to have HCU, stratifications by tHcy levels, clinical characteristics, and phenotypic expressions were applied to refine the cohort. Included patients were then stratified by highest tHcy level. Clinical burden was measured by category of HCU-related events. Descriptive statistics were reported.

**Results:**

Six hundred thirty-three patients met the inclusion criteria, and 601 patients had a tHcy level: < 50 µM (n = 278), 50 to < 100 µM (n = 212), and ≥ 100 µM (n = 111). Among the 601 patients with a tHcy level, almost one-half (n = 297, 49.4%) had at least one thrombotic/thromboembolic, skeletal, ocular, or neurological event and 14.1% (n = 85) had multiple events. Thrombotic/thromboembolic events (n = 186, 30.9%) were the most common type of events, followed by skeletal (n = 100, 16.6%), ocular (n = 63, 10.5%), and neurological events (n = 50, 8.3%). During follow-up, 5.7% (n = 34) of the patients died. All events assessed were more prevalent in the 50 to < 100 µM group and ≥ 100 µM group compared with those in the < 50 µM group.

**Conclusions:**

As has been believed, patients with tHcy ≥ 100 µM carried a substantial clinical burden, but the burden is also very high in those whose levels were ≥ 50 µM. Thrombotic/thromboembolic events were more common than skeletal, ocular, or neurological events. Meaningfully lowered tHcy levels may help to reduce significant clinical events.

**Supplementary Information:**

The online version contains supplementary material available at 10.1186/s13023-025-03530-9.

## Background

Classical homocystinuria (HCU) is a rare genetic metabolic disorder that is caused by pathogenic variants in the cystathionine beta-synthase (*CBS*) gene and characterized by elevated homocysteine (Hcy) and methionine (Met) levels [1–3].

The clinical presentation of HCU is variable, including age at which it is diagnosed, occurrence of complications, and severity of those complications. HCU-related complications can include thrombotic/thromboembolic events, skeletal changes such as pectus excavatum, marfanoid habitus, and low bone mineral density, ocular complications such as ectopia lentis and myopia, and neurological disease including seizures, cognitive impairment, and developmental delays [2, 4]. However, HCU-related complications occur in varying degrees and patients can have one or all organs/systems affected [4]. Additionally, these complications do not always begin in childhood and complications can first appear in patients as adults [4].

The exact prevalence of HCU is not well defined but has been reported to be from 1:200,000 to 1:335,000 worldwide [4]. Factors that contribute to the difficulty establishing an exact prevalence estimate include potential missed diagnoses on newborn screens [5] and difficulty with clinical diagnosis due to the wide spectrum of potential complications [6].

While complex, the aim of treatment for HCU is to reduce plasma total Hcy (tHcy) to a safe level, with a target of < 100 μM for patients with pyridoxine-unresponsive HCU and < 50 μM for patients with pyridoxine-responsive HCU [1]. Treatment can include dietary modification with a Met-restricted diet (a low-protein diet supplemented with a Met-free amino acid formula) and pharmacologic treatment (such as with pyridoxine, if responsive, adjunctive betaine, and vitamin B12 and folate supplementation) [1, 7, 8]. Achieving and maintaining safe tHcy levels is often difficult unless treatment begins during infancy and adherence is achieved throughout the lifetime of a patient [2, 9]. The restrictive, lifelong dietary modifications play a significant role in this difficulty with adherence [1, 10].

Classical homocystinuria has been known and studied for over 50 years [2] and the clinical characteristics and associated complications of HCU well documented [1, 2, 4]. However, the clinical burden is not well quantified as a result of the rare nature of the disease and broad clinical features. Additionally, there is limited published research examining the association between the frequency of the broad spectrum of complications and tHcy levels, especially using claims data [11]. In addition to a study by Mudd et al. [2], previous research on this topic has mainly been small sample observational studies on a specific clinical area with limited focus on stratification by tHcy levels [12–16].

This study aimed to describe the overall clinical burden of patients with HCU in the United States (US) and key clinical events by tHcy levels using administrative claims data. This study is one of the first claims-based burden studies that stratifies events by tHcy levels.

## Methods

This was a non-interventional, retrospective cohort analysis in the US, with a study period from January 01, 2016, through September 30, 2021.

### Data source

This study used Optum’s de-identified Market Clarity Data (Market Clarity), which links electronic health record (EHR) data with historical, linked administrative claims data, pharmacy claims, physician claims, and facility claims (with clinical information) and is inclusive of medications prescribed and administered. Clinically rich and specific data elements sourced from the EHR include lab results, vital signs and measurements, diagnoses, procedures, and information derived from unstructured clinical notes using natural language processing (NLP).

Optum data only contains de-identified health information as described by the Health Insurance Portability and Accountability Act (HIPAA) Privacy Rule. No direct identifiers of individuals, employers, households, or providers are included. The data are de-identified in accordance with the HIPAA Privacy Rule's de-identification standard. Optum uses proprietary processes for record/data anonymization and privacy protection. Based on US federal regulations (45 CRF 46.104 – Exempt Research), we believe this study is exempt from Institutional Review Board review. Since Optum data are de-identified and its use does not require Institutional Review Board approval, it typically does not require patient consent.

### Study population and participants

**Fig. 1:**
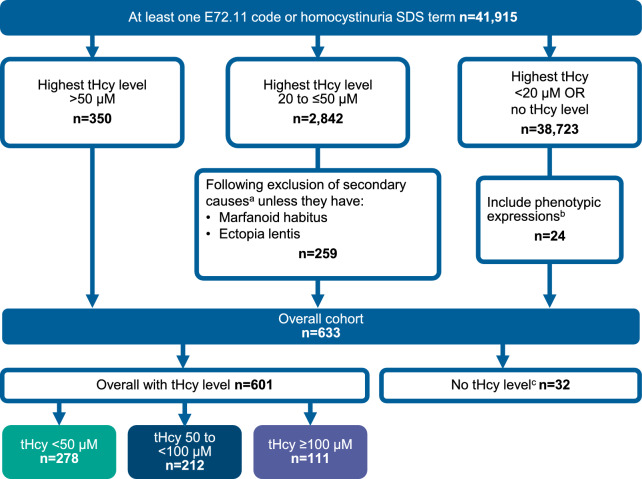
Classical Homocystinuria Patient Identification Algorithm. CKD, chronic kidney disease; ESKD, end-stage kidney disease; MI, myocardial infarction; mo, month; SDS, signs, disease, and symptoms; tHcy, total homocysteine. ^a^Secondary causes; At any time: Megaloblastic anemia, disorder of cobalamin metabolism, folate deficiency, CKD, ESKD, renal transplant, diabetes, hypothyroidism; Within 12 mo: MI. ^b^Phenotypic expressions: 1. Ectopia lentis AND (cerebrovascular thrombotic/thromboembolic event OR neurologic feature) exclude: Marfanoid habitus, sulfite oxidase deficiency (E72.19); 2. Pectus excavatum AND (cerebrovascular thrombotic/thromboembolic event OR [any thrombotic/thromboembolic event AND neurologic feature]) exclude: Marfanoid habitus, sulfite oxidase deficiency (E72.19); 3. Marfanoid habitus AND cerebrovascular thrombotic/thromboembolic event AND neurologic feature AND (ectopia lentis OR pectus excavatum) exclude: Sulfite oxidase deficiency (E72.19). ^c^Patients with outlier (≥ 3,000 µM) tHcy levels only (no other tHcy level) were considered as having no tHcy level for the stratifications and thus excluded from assessment of the tHcy subgroups

Patients were included in this study if they had 1 or more International Classification of Diseases, Tenth Revision (ICD-10) code for homocystinuria (E72.11) or the signs, disease, and symptoms (SDS) term *homocystinuria* in the NLP dataset. An ICD-10 code specific to HCU does not exist, and no other terms related to homocystinuria were found in the NLP dataset. Therefore, in order to obtain a study population most likely to have HCU, tHcy levels, clinical characteristics, and phenotypic expressions were all applied to refine the cohort. Patients with tHcy > 50 μM were included with no other restrictions. In patients with tHcy 20 to ≤ 50 μM, those with secondary causes of elevated tHcy were excluded unless they had other clinical presentations indicative of HCU (Fig. 1). In patients with tHcy < 20 µM or no tHcy level, patients were excluded unless they had phenotypical expressions of HCU (Fig. 1). The index date used was the date on which the first criterion for inclusion was met, within the identification period. All available data prior to and after the index date were used.

### Variables and outcomes

Patients who met the inclusion criteria were stratified by highest tHcy levels (< 50 µM, 50 to < 100 µM, ≥ 100 µM, and no level) during the study period. Extremely high levels (≥ 3,000 µM) were considered outliers and excluded from the tHcy stratifications as unexpectedly high levels are likely due to testing or data entry errors. Baseline demographics and clinical characteristics were assessed. These included index year, age at index (in years), age group at index, gender, region, race, health insurance type, follow-up time (in months), Charlson comorbidity index (CCI), HCU-related Charlson comorbidities, highest tHcy level, and highest Met level (at any time during the study period).

Clinical burden was measured by assessing clinical events by category of HCU-related events and individually by specific clinical event. HCU-related event categories included thrombotic/thromboembolic, skeletal, ocular, and neurological events. Thrombotic/thromboembolic events included deep vein thrombosis (DVT), stroke/transient ischemic attack (TIA), and related conditions. Skeletal events included osteoporosis, pectus excavatum, pectus carinatum, fractures, and related conditions. Ocular events included retinal detachment, lens dislocation, myopia, and related conditions. Neurological events included seizure disorder/epilepsy, hemiplegia/hemiparesis, and related conditions. See Additional file 2 for a full list of ICD-10 codes used to identify the clinical events. Major clinical events were defined as having at least 1 condition-related emergency department (ED) or outpatient visit or at least 1 inpatient admission. Event rates per 10,000 person-years were also reported.

In addition, all-cause mortality and clinical event combinations were assessed including any HCU-related event and multiple HCU-related events (defined as patients who experienced more than 1 of the HCU-related event categories listed above). All-cause mortality was defined as having a record of death during follow-up. Patients were followed from the index date to the end of their data availability or to the end of the study period, whichever occurred earlier.

### Statistical analysis

Patient demographics, clinical characteristics, and clinical events were summarized using descriptive statistics. Categorical variables were summarized using frequencies and percentages. Continuous variables were summarized using means (standard deviations [SDs]) and percentiles (minimum, 1st quartile, median, 3rd quartile, and maximum). Event rates per 10,000 person-years were calculated as the number of condition-related ED, outpatient, or inpatient visits with a primary diagnosis per 10,000 person-years during follow-up. Linear regression with a generalized estimating equation and the Jonckheere-Terpstra test were used to assess trends across the tHcy levels. Patients without tHcy levels were not included in statistical comparisons.

## Results

### Patient demographics and clinical characteristics

There were approximately 71 million patients in Market Clarity during the study period. Of those, 41,915 had at least 1 ICD-10 code for homocystinuria (E72.11) or SDS NLP term for homocystinuria in the dataset. Among those, 350 patients had a tHcy level above 50 µM. There were 2,842 patients with a tHcy level between 20 and 50 µM and after excluding secondary causes, 259 patients remained in this category. There were 38,723 patients with tHcy level < 20 µM or no tHcy level and after only including those with phenotypic expressions, 24 patients remained. Following the patient identification algorithm (Fig. 1) there were 633 patients meeting the inclusion criteria. Among those, 601 patients had a tHcy level available and were split into 3 patient groups based on their highest tHcy level: < 50 µM (n = 278), 50 to < 100 µM (n = 212), and ≥ 100 µM (n = 111). Thirteen patients were excluded due to outlier tHcy levels (≥ 3,000 µM).

In the overall cohort (n = 633), approximately one-half of the patients were female (46.6%), one-half were from the Midwest (54.3%), and a majority of the patients were White (79.3%) (Table 1). Mean (SD) age was 50.0 (18.0) years and most patients were between 18 and 64 years old (72.5%) (Table 1). Approximately one-half (49.8%) of patients had commercial insurance and nearly one-fourth had Medicaid or Medicare insurance (23.1% and 22.9%, respectively) (Table 1). Mean (SD) follow-up time was 30.9 (19.4) months and mean (SD) CCI was 1.1 (1.8) (Table 1).

**Table 1:** Baseline Demographics and Clinical Characteristics, Stratified by Highest Total Homocysteine Level During the Study Period

			By highest tHcy level			
	Overall cohort	Overall with tHcy level	< 50 µM	50 to < 100 µM	≥ 100 µM	No tHcy level
Total	633	601	278	212	111	32
*Index year, No. (%)*						
2016	188 (29.7)	175 (29.1)	74 (26.6)	53 (25.0)	48 (43.2)	13 (40.6)
2017	156 (24.6)	145 (24.1)	75 (27.0)	53 (25.0)	17 (15.3)	11 (34.4)
2018	122 (19.3)	116 (19.3)	60 (21.6)	40 (18.9)	16 (14.4)	6 (18.8)
2019	84 (13.3)	83 (13.8)	31 (11.2)	36 (17.0)	16 (14.4)	1 (3.1)
2020	59 (9.3)	58 (9.7)	28 (10.1)	22 (10.4)	8 (7.2)	1 (3.1)
2021 (up to September)	24 (3.8)	24 (4.0)	10 (3.6)	8 (3.8)	6 (5.4)	0 (0.0)
*Age at index (continuous)*						
Mean (SD)	50.0 (18.0)	49.7 (18.0)	54.0 (16.2)	48.8 (17.0)	40.8 (20.8)	55.0 (17.0)
Median (Q1, Q3)	51.0 (39.0, 63.0)	50.0 (39.0, 62.0)	55.0 (44.0, 66.0)	48.0 (40.0, 61.0)	42.0 (25.0, 54.0)	53.0 (41.0, 71.5)
Min–Max	0–87	0–87	0–87	0–86	0–86	26–83
*Age (categorical), No. (%)*						
< 18	33 (5.2)	33 (5.5)	5 (1.8)	12 (5.7)	16 (14.4)	0 (0.0)
18–44	192 (30.3)	181 (30.1)	66 (23.7)	71 (33.5)	44 (39.6)	11 (34.4)
45–64	267 (42.2)	256 (42.6)	130 (46.8)	93 (43.9)	33 (29.7)	11 (34.4)
65–74	92 (14.5)	88 (14.6)	51 (18.3)	25 (11.8)	12 (10.8)	4 (12.5)
≥ 75	49 (7.7)	43 (7.2)	26 (9.4)	11 (5.2)	6 (5.4)	6 (18.8)
*Gender, No. (%)*						
Female	295 (46.6)	277 (46.1)	122 (43.9)	105 (49.5)	50 (45.0)	18 (56.3)
Male	338 (53.4)	324 (53.9)	156 (56.1)	107 (50.5)	61 (55.0)	14 (43.8)
*Region, No. (%)*						
Midwest	344 (54.3)	319 (53.1)	151 (54.3)	116 (54.7)	52 (46.8)	25 (78.1)
Northeast	86 (13.6)	85 (14.1)	37 (13.3)	27 (12.7)	21 (18.9)	1 (3.1)
South	24 (3.8)	22 (3.7)	12 (4.3)	7 (3.3)	3 (2.7)	2 (6.3)
West	96 (15.2)	93 (15.5)	40 (14.4)	35 (16.5)	18 (16.2)	3 (9.4)
Other/Unknown	83 (13.1)	82 (13.6)	38 (13.7)	27 (12.7)	17 (15.3)	1 (3.1)
*Race, No. (%)*						
African American	80 (12.6)	75 (12.5)	27 (9.7)	37 (17.5)	11 (9.9)	5 (15.6)
Asian	9 (1.4)	9 (1.5)	2 (0.7)	5 (2.4)	2 (1.8)	0 (0.0)
White	502 (79.3)	475 (79.0)	227 (81.7)	160 (75.5)	88 (79.3)	27 (84.4)
Other/Unknown	42 (6.6)	42 (7.0)	22 (7.9)	10 (4.7)	10 (9.0)	0 (0.0)
*Insurance type, No. (%)*						
Commercial	315 (49.8)	297 (49.4)	148 (53.2)	100 (47.2)	49 (44.1)	18 (56.3)
Medicaid	146 (23.1)	143 (23.8)	42 (15.1)	63 (29.7)	38 (34.2)	3 (9.4)
Medicare	145 (22.9)	135 (22.5)	77 (27.7)	40 (18.9)	18 (16.2)	10 (31.3)
Other payor type	8 (1.3)	8 (1.3)	5 (1.8)	2 (0.9)	1 (0.9)	0 (0.0)
Uninsured	12 (1.9)	11 (1.8)	5 (1.8)	3 (1.4)	3 (2.7)	1 (3.1)
Unknown	7 (1.1)	7 (1.2)	1 (0.4)	4 (1.9)	2 (1.8)	0 (0.0)
*Follow-up time, months*^*a*^						
Mean (SD)	30.9 (19.4)	30.8 (19.5)	30.3 (19.3)	30.4 (18.7)	32.8 (21.4)	33.5 (18.0)
Median (Q1, Q3)	29.5 (14.4, 45.5)	29.2 (14.2, 45.5)	30.0 (13.0, 45.4)	27.8 (15.9, 44.2)	31.9 (14.4, 49.3)	38.0 (18.7, 45.8)
Min–Max	0–69.8	0–69.8	0–69.6	0–69.2	0–69.8	0–69.4
*Charlson comorbidity index*						
Mean (SD)	1.1 (1.8)	1.1 (1.7)	0.8 (1.4)	1.3 (1.8)	1.3 (2.0)	1.8 (2.9)
Median (Q1, Q3)	0.0 (0.0–2.0)	0.0 (0.0–2.0)	0.0 (0.0–1.0)	1.0 (0.0–2.0)	0.0 (0.0- 2.0)	1.0 (0.0- 2.0)
Min–Max	0–15	0–15	0–11	0–15	0–11	0–12
*Charlson comorbidities, No. (%)*^*b*^						
Cerebrovascular disease	110 (17.4)	102 (17.0)	35 (12.6)	49 (23.1)	18 (16.2)	8 (25.0)
Peripheral vascular disease	99 (15.6)	93 (15.5)	34 (12.2)	45 (21.2)	14 (12.6)	6 (18.8)
Congestive heart failure	48 (7.6)	43 (7.2)	11 (4.0)	22 (10.4)	10 (9.0)	5 (15.6)
Hemiplegia or paraplegia	40 (6.3)	38 (6.3)	14 (5.0)	16 (7.5)	8 (7.2)	< 5
Myocardial infarction	27 (4.3)	27 (4.5)	8 (2.9)	12 (5.7)	7 (6.3)	0 (0.0)
Dementia	8 (1.3)	8 (1.3)	< 5	0 (0.0)	< 5	0 (0.0)

*Max* Maximum, *Min* Minimum, *Q1* 1st quartile, *Q3* 3rd quartile, *SD* Standard deviation, *tHcy* Total homocysteine

^a^Time based on activity in electronic health record dataset during period of interest

^b^The Optum minimum sample requirement is 5 or more subjects or patients for publications. Per guidance provided by Optum, all results indicated < 5 are not shown due to small cell sizes

Demographic trends in all the groups by tHcy levels were similar (Table 1). However, in the patients with tHcy ≥ 100 µM the mean age was slightly lower at 40.8 years old, there was a higher proportion of patients less than 18 years old (14.4% vs less than 6.0% in the other categories), and there was a lower proportion of patients 45–64 years old (29.7% vs slightly above 40.0% in the other categories) (Table 1).

Among the patients with a tHcy level available (n = 601), the mean (SD) highest tHcy level was 68.4 (77.1) µM (Table 2). Mean (SD) highest tHcy in patients in the < 50 µM group was 25.8 (7.6) µM (Table 2). For patients in the 50 to < 100 µM group, mean (SD) highest tHcy was 66.6 (13.2) µM and for patients in the ≥ 100 µM group, mean (SD) highest tHcy was 178.6 (122.9) µM (Table 2). Among patients with a tHcy level available, 2.7% (n = 16) had a highest tHcy < 20 µM, 43.6% (n = 262) had a highest tHcy level of 20 to < 50 µM, 35.3% (n = 212) had a highest tHcy of 50 to < 100 µM, and 18.5% (n = 111) had a highest tHcy ≥ 100 µM (Table 2).

**Table 2:** Maximum Homocysteine Levels, Stratified by Highest Total Homocysteine Level During the Study Period^a^

	Overall with tHcy level	< 50 µM	50 to < 100 µM	≥ 100 µM
Patients with ≥ 1 lab, No. (%)	601 (100.0)	278 (100.0)	212 (100.0)	111 (100.0)
Mean (SD), µM	68.4 (77.1)	25.8 (7.6)	66.6 (13.2)	178.6 (122.9)
Median (Q1, Q3), µM	52.0 (24.1, 78.4)	23.6 (21.0, 28.0)	64.2 (55.0, 74.3)	137.1 (119.0, 184.7)
Min–Max, µM	4–877	4–49.9	50–99.3	100–877
*Highest tHcy level, categorical, No. (%)*				
< 20 µM	16 (2.7)	16 (5.8)	N/A	N/A
20 to < 50 µM	262 (43.6)	262 (94.2)	N/A	N/A
≥ 50 µM	323 (53.7)	N/A	212 (100.0)	111 (100.0)
50 to < 100 µM	212 (35.3)	N/A	212 (100.0)	N/A
≥ 100 µM	111 (18.5)	N/A	N/A	111 (100.0)

*Max* Maximum, *Min* Minimum, *N/A* Not applicable, *Q1* 1st quartile, *Q3* 3rd quartile, *SD* Standard deviation, *tHcy* Total homocysteine

^a^At any time during the study period

### Clinical events in patients in the overall cohort with a total homocysteine level available

**Fig. 2:**
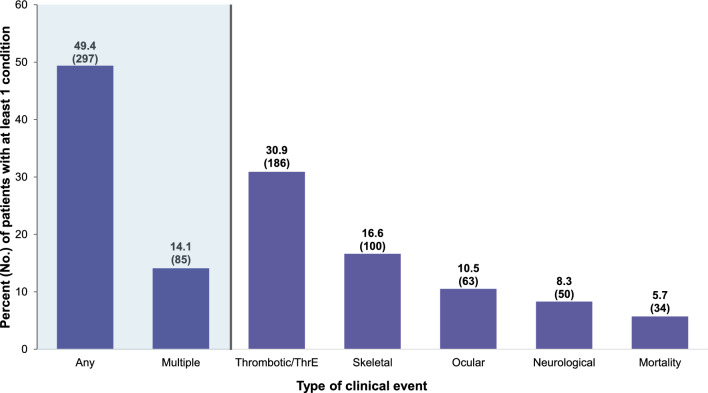
Clinical Event Categories in Patients in the Overall Cohort With a Total Homocysteine Level. HCU, classical homocystinuria; ThrE, thromboembolic. Any event includes any thrombotic/ThrE, skeletal, ocular, or neurological events. Multiple events include at least 2 HCU-related events. Patients can have multiple individual components of clinical events. n = 601

Among patients with a tHcy level available (n = 601), almost one-half (n = 297, 49.4%) of patients had at least one thrombotic/thromboembolic, skeletal, ocular, or neurological event and 14.1% (n = 85) had multiple events (Table 3, Fig. 2). Thrombotic/thromboembolic events (n = 186, 30.9%) were the most common type of events, followed by skeletal (n = 100, 16.6%), ocular (n = 63, 10.5%), and neurological events (n = 50, 8.3%) (Table 3, Fig. 2). During follow-up, 5.7% (n = 34) of the patients died (Table 3, Fig. 2).

**Table 3:** Clinical Event Categories in Patients With Classical Homocystinuria, by Highest Total Homocysteine Level

			By highest tHcy level				
	Overall cohort	Overall with tHcy level	< 50 µM	50 to < 100 µM	≥ 100 µM	No tHcy level	*p* values^c,d^
Total	633	601	278	212	111	32	
*Thrombotic/thromboembolic*							
≥ 1 condition, No. (%)	203 (32.1)	186 (30.9)	62 (22.3)	85 (40.1)	39 (35.1)	17 (53.1)	< 0.001
Event rate per 10,000 person-years^a^ (rate, SE)	12,593, 280	12,604, 288	7,021, 319	21,903, 643	9,002, 549	12,399, 1,182	
*Skeletal*							
≥ 1 condition, No. (%)	107 (16.9)	100 (16.6)	41 (14.7)	38 (17.9)	21 (18.9)	7 (21.9)	0.50
Event rate per 10,000 person- years^a^ (rate, SE)	2,803, 132	2,789, 136	2,157, 177	3,808, 268	2,443, 286	3,044, 586	
*Ocular*							
≥ 1 condition, No. (%)	70 (11.1)	63 (10.5)	27 (9.7)	24 (11.3)	12 (10.8)	7 (21.9)	0.82
Event rate per 10,000 person- years^a^ (rate, SE)	1,666, 102	1,566, 102	1,737, 159	999, 137	2,175, 270	3,382, 617	
*Neurological*							
≥ 1 condition, No. (%)	56 (8.8)	50 (8.3)	16 (5.8)	20 (9.4)	14 (12.6)	6 (18.8)	0.06
Event rate per 10,000 person- years^a^ (rate, SE)	2,592, 127	2,526, 129	2,215, 179	1,828, 186	4,484, 387	3,720, 648	
*Mortality*							
Died, No. (%)	34 (5.4)	34 (5.7)	7 (2.5)	16 (7.5)	11 (9.9)	0 (0.0)	0.004
Death rate per 10,000 person- years^a^ (rate, SE)	211, 36	224, 38	101, 38	302, 75	368, 111	0, 0	
*Event combinations, No. (%*)							
Patients with any thrombotic/thromboembolic, skeletal, ocular, neurological event	319 (50.4)	297 (49.4)	114 (41.0)	121 (57.1)	62 (55.9)	22 (68.8)	< 0.001
Patients with multiple events^b^	95 (15.0)	85 (14.1)	27 (9.7)	37 (17.5)	21 (18.9)	10 (31.3)	0.012
Patients with any event	429 (67.8)	404 (67.2)	173 (62.2)	154 (72.6)	77 (69.4)	25 (78.1)	0.045
Patients with multiple events^b^	218 (34.4)	200 (33.3)	70 (25.2)	86 (40.6)	44 (39.6)	18 (56.3)	< 0.001

*ED* Emergency department, *SE* Standard error, *tHcy* Total homocysteine

^a^Defined as the number of condition-related ED or outpatient visits, or inpatient admissions with a primary diagnosis per 10,000 person-years during follow-up

^b^Patients with multiple events were defined as those with at least 2 different types of listed events

^c^Comparison was assessed using linear regression with a generalized estimating equation and the Jonckheere-Terpstra test for continuous outcomes (means and medians, respectively), and Fisher’s exact test for categorical variables if a cell has a 0 value or frequency is ≤ 5, or Chi-square test

^d^The no tHcy level subgroup was not included in the statistical test comparison

Rates of HCU-related clinical events in our study population compared with the general population are shown in Table 4. In our study, among patients who had a thrombotic/thromboembolic event (n = 186), stroke/TIA was the most common (n = 87, 46.8%), with DVT (n = 58, 31.2%) and pulmonary embolism (PE) (n = 46, 24.7%) second and third most common, respectively (Supplementary Table 1 [see Additional file 1]). Among patients who had a skeletal event (n = 100), fracture (n = 66, 66.0%) was the most common and osteoporosis (n = 20, 20.0%) was the second most common (Supplementary Table 1 [see Additional file 1]). Among patients who had an ocular event (n = 63), glaucoma (n = 26, 41.3%) was most common, followed by myopia (n = 19, 30.2%), and lens dislocation (n = 13, 20.6%) (Supplementary Table 1 [see Additional file 1]). Among patients who had a neurological event (n = 50), epilepsy (n = 31, 62.0%) was the most common, followed by hemiplegia/hemiparesis (n = 12, 24.0%) (Supplementary Table 1 [see Additional file 1]).

**Table 4:** Prevalence Rate of Clinical Events in the Overall Cohort and in the General Population

	Overall cohort with tHcy level (n = 601)	General population
Fractures, %	11.0	7.4^a^
Stroke/Transient ischemic attack, %	14.5	3.3^b^
Epilepsy, %	5.2	1.2^c^
Deep vein thrombosis or pulmonary embolism, %	17.3	0.3^d^
Ectopia lentis, %	2.2	0.006^e^
Osteoporosis, %	3.3	12.6^f^

*tHcy* Total homocysteine

^a^Calculated by major osteoporotic fracture probability in adults 50 and over [17]

^b^Prevalence of stroke only, not including transient ischemic attack. From 2017 to 2020, ≥ 20 years of age [18]

^c^Prevalence of active epilepsy [19]

^d^Calculated based on 900,000 people with deep vein thrombosis or pulmonary embolism divided by the United States population, using 331,900,000 [20]

^e^Based on a study in Denmark [21]

^f^Prevalence of osteoporosis in adults 50 and over [22]

### Clinical events in patients stratified by highest total homocysteine level

**Fig. 3:**
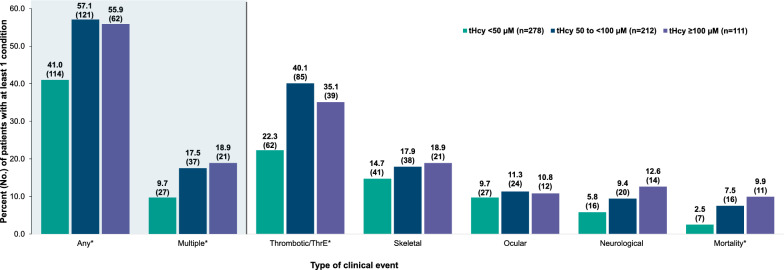
Clinical Event Categories in Patients With Classical Homocystinuria by Highest Total Homocysteine Level. HCU, classical homocystinuria; tHcy, total homocysteine; ThrE, thromboembolic. *Event categories with p < 0.05. Any event includes any thrombotic/ThrE, skeletal, ocular, or neurological events. Multiple events include at least 2 HCU-related events

**Fig. 4:**
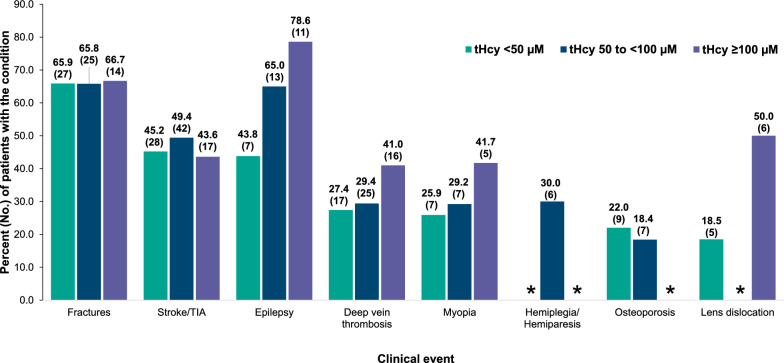
Clinical Events in Patients with Classical Homocystinuria by Highest Total Homocysteine Level. tHcy, total homocysteine; TIA, transient ischemic attack. The denominator for percentages is the total number of patients with each type of event (ocular, skeletal, etc.) within each tHcy group. p values are not included due to censoring of small sample sizes. *The Optum minimum sample requirement is 5 or more subjects or patients for publications. Per guidance provided by Optum, all results indicated as < 5 are not shown due to small cell sizes

All events assessed were more prevalent in the groups with higher tHcy levels, particularly when comparing patients in the 50 to < 100 µM group and ≥ 100 µM group with those in the < 50 µM group. The proportion of patients in the clinical event categories was similar for patients in the tHcy of 50 to < 100 µM group and those in the ≥ 100 µM group (Table 3, Fig. 3). However, there were slightly higher proportions of patients in the ≥ 100 µM group compared with patients in the 50 to < 100 µM and < 50 µM groups in the multiple (18.9%, 17.5%, and 9.7%, respectively), skeletal (18.9%, 17.9%, and 14.7%, respectively), neurological (12.6%, 9.4%, and 5.8%, respectively), and mortality (9.9%, 7.5%, and 2.5%, respectively) event categories (Table 3, Fig. 3). A higher proportion of patients in the ≥ 100 µM and the 50 to < 100 µM groups were found to have epilepsy compared with patients in the < 50 µM group (78.6%, 65.0% and 43.8%, respectively) (Supplementary Table 1 [see Additional file 1], Fig. 4). A higher proportion of patients in the ≥ 100 µM group were found to have DVT (41.0%), myopia (41.7%), and lens dislocation (50.0%), compared with patients in the < 50 µM group (27.4%, 25.9%, 18.5%, respectively) (Supplementary Table 1 [see Additional file 1], Fig. 4).

### Event rates per 10,000 person-years in the overall cohort and stratified by highest total homocysteine level

Event rates per 10,000 person-years were high in the overall cohort and across tHcy groups. The highest event rates for thrombotic/thromboembolic and skeletal events were seen in the patients in the 50 to < 100 µM group (21,903 and 3,808, respectively) (Table 3). The highest event rates for ocular and neurological events were seen in patients in the ≥ 100 µM group (2,175 and 4,484, respectively) (Table 3).

## Discussion

This real-world study of health insurance claims data assessed the overall clinical burden of patients with HCU and stratified key clinical events by tHcy level in this patient population. It is one of the first claims-based burden studies to do so. Close to one-half of patients experienced at least one major HCU-related clinical event and 14% had more than 1 event throughout the follow-up period of close to 2.5 years. In our study, thrombotic/thromboembolic events were more common than skeletal, ocular, or neurological events. The clinical burden was found to be substantial, particularly in patients with tHcy levels at or above 50 μM. Clinical event rates were generally higher in the 50 to < 100 µM and ≥ 100 μM groups compared with the < 50 µM group. Mortality rates were also higher in patients with higher tHcy levels.

Additionally, other than osteoporosis, the rates of HCU-related clinical events assessed in our study population were found to be higher than those in the general population. These differences were most notable in the rates of ectopia lentis (2.2% in our study compared with 0.006% in the general population) [21], DVT or PE (17.3% in our study compared with 0.3% in the general population) [20], stroke/TIA (14.5% compared with 3.3% in the general population) [18], and epilepsy (5.2% compared with 1.2% in the general population) [19]. Osteoporosis rates in the general population are more difficult to determine as it is an age-related disease. As a result, this limits comparison with our study population. Overall, these comparisons to the general population provide additional context for the high clinical burden seen in patients with HCU in our study.

The demographics of the patient population in our study are generally consistent with other similar database or dataset studies [23, 24]. Any slight differences observed are likely due to variations in data sources. Interestingly, in our study we found that patients with higher tHcy levels (≥ 100 µM) had a lower mean age and a higher proportion of patients less than 18 years old, compared with patients with lower tHcy levels (< 100 µM). This could be a result of patients with more severe HCU being diagnosed at younger ages and those with less severe HCU and less HCU-related complications being diagnosed later in life or undiagnosed. Additionally, increased mortality rate in patients with more severe disease could result in fewer patients reaching older age.

The clinical burden of HCU has been described in several observational cohort studies. In contrast to what we observed in our study, a Brazilian study of 72 patients (of which 44 had current clinical data available at the time of study inclusion) by Poloni et al. [25] found ocular events to occur at a higher frequency (93%), followed by neurological (70%), skeletal (61%), and then vascular manifestations (25%) [25]. A retrospective study of 25 patients by Almuqbil et al. [14] that assessed the prevalence of psychological symptoms in patients with HCU also reported that ectopia lentis was the most common complication in their study (n = 11, 44%), followed by osteoporosis (n = 7, 28%) [14].

While similar, but also slightly lower compared with what we observed, a study by Mudd et al. [2] that compiled data on 629 patients with CBS deficiency via an international survey found thromboembolic events to occur in 25% of patients with HCU [2]. In their study, just over one-half of thromboembolic events affected peripheral veins (with one-fourth resulting in a PE) [2]. The study by Mudd et al. [2] found fewer rates of cerebrovascular accidents (32%) [2], but did not specify if TIA was included in this category as was in our study. This could explain the higher rates noted in our study.

While epilepsy was overall the most common type of neurological disease in our study, we observed fewer than the approximately 21% of patients who were reported to have had seizures in the study by Mudd et al. [2].

Comparisons with the aforementioned studies and our study are limited for a variety of reasons. Ectopia lentis tends to occur at a younger age while thrombotic/thromboembolic events tend to occur at an older age. This could explain why rates of ectopia lentis were lower and rates of thrombotic/thromboembolic events were slightly higher in our study population, given the higher median age in our study compared with the studies by Polini et al. [25] and Almuqbil et al. [14]. Ectopia lentis was a criterion for acceptance into the Mudd et al. study [2] which limits comparisons. Additionally, severity of the disease could play a role in some of the differences noted. Close to 85% of the patients from the Poloni et al. [25] study and 80% from the Almuqbil et al. [14] study were nonresponsive to pyridoxine. This could affect rates of complications. Another important difference is the shorter length of follow-up data in our study. Since our study looked at a 5-year window of time, it is not directly comparable with other studies that are fully retrospective and include all diagnoses a patient has had in their lifetime. Additionally, within our analysis it is possible that previous diagnoses were made and not captured given constraints of the dataset. The above studies also did not stratify patients by tHcy level, which may be an important factor in determining clinical burden in patients with HCU. Lastly, differences in sample sizes and geographic variances (such as in prevalence, healthcare, and newborn screening) could also explain some of the differences noted.

These study/survey results highlight why the clinical burden of thrombotic/thromboembolic events is an important clinical event to assess. Patients with HCU can remain undiagnosed into adulthood and then develop thrombotic/thromboembolic events as their only symptom [6]. Also, it has been shown that treatment of HCU decreases the incidence of thrombotic/thromboembolic events if tHcy levels are reduced, even if tHcy levels are not normalized [26]. The results of our study provide support for early diagnosis and treatment of HCU to prevent/mitigate the significant morbidity and mortality that thrombotic/thromboembolic events can cause in patients with HCU [4, 27].

Published data have suggested that complications of HCU are unlikely if the tHcy level of patients is less than 120 µM [1, 28]. In our study we found that clinical event rates were generally higher in patients in the 50 to < 100 µM and ≥ 100 μM groups compared with patients in the < 50 µM group. This study corroborates and supports what is already known regarding the pattern of clinical events in these patients. However, our study also highlights the potential for substantial clinical burden in patients with tHcy of 50 to < 100 µM, particularly for thrombotic/thromboembolic events and epilepsy. While our study did not assess causality, we found that tHcy levels less than 50 µM aligned with fewer clinical events. This suggests there could be a potential association between maintaining tHcy levels less than 50 µM and a decreased clinical burden, indicating a potential benefit from dietary and/or pharmacologic treatments aimed at lowering tHcy to these levels. Standard practice has been to maintain tHcy levels below 100 µM [1]. However, with continued research this threshold could potentially be lowered to below 50 µM. Future studies could also look at the impact other variables, such as lifestyle choices and long-term treatment effects, have on the clinical burden and outcome of patients with HCU.

### Limitations

Limitations associated with real-world dataset studies apply to this analysis. The results of our study are mainly generalizable to a commercially insured population in the Midwest US, consistent with Market Clarity used for the study. If patients had undiagnosed diseases or had diagnoses outside the database network, they would not be able to be ascertained for this study. Additionally, there was a decrease in the number of patients available within the dataset over time, potentially due to the COVID pandemic that occurred during the study period. There was also limited availability of tHcy test results and an additional limitation was our reliance on the highest tHcy levels for any given patient. Mortality results should be interpreted with caution due to the limitations in capturing mortality events in the dataset used for the study.

An ICD-10 diagnosis code does not exist for HCU. As such, identifying patients with HCU using claims data is difficult. The patient identification algorithm used in this study aimed to accurately identify patients with HCU using diagnosis codes, lab values, and clinical presentations via a tiered approach. However, due to limitations in the dataset, confirmation of a diagnosis of HCU was not always possible. It is also important to note that patients with HCU could be excluded as a result of self-management with vitamin intake or diet.

Additionally, the higher age in our study population could impact the event rates in diseases where age is a risk factor, such as cardiovascular disease.

## Conclusion

This study assessed the overall clinical burden of patients with HCU and stratified key clinical events by tHcy level in this patient population. Overall, the clinical burden of patients with HCU appears to be substantial. As has been believed, patients with tHcy ≥ 100 µM carried a substantial burden, but the burden was also very high in those whose levels were ≥ 50 µM. Thrombotic/thromboembolic events were more common than skeletal, ocular, or neurological events. The results of our retrospective observational study suggest that for patients with HCU, meaningfully lowered tHcy should result in prevention/reduction of significant clinical events. Standard practice based on current guidelines has been to maintain tHcy levels below 100 µM [1], with little to no guidance available on how patients with tHcy levels between 50 µM and 100 µM should be managed. Should our findings be corroborated with additional confirmatory evidence in the future, a case could be made for clinical guidelines to recommend lowering the threshold for maintenance tHcy levels from below 100 µM to below 50 µM.

## Supplementary Information

Additional file 1.

Additional file 2.

## Data Availability

The data that support the findings of this study are available from Optum (https://business.optum.com/en/data-analytics/life-sciences/real-world-data/market-clarity-data.html), but restrictions apply to the availability of these data, which were used under license for the current study, and so are not publicly available. Data are however available from the authors upon reasonable request and with permission of Optum.
