# Supplementary material for: High clinical burden of classical homocystinuria in the United States: a retrospective analysis
Source: Orphanet J Rare Dis. 2025 Jan 24;20:37. doi: 10.1186/s13023-025-03530-9 (PMC11762879; doi:10.1186/s13023-025-03530-9)
Supplement: Supplementary file 1 — Additional file 1. [file 13023_2025_3530_MOESM1_ESM.docx]

**Supplementary Material**

**Supplementary Table 1. Clinical Events in Patients With Classical Homocystinuria by Highest Total Homocysteine Level**

|  | **By highest tHcy levels** | | | | | | | |
| --- | --- | --- | --- | --- | --- | --- | --- | --- |
|  | **Overall cohort** | **Overall with tHcy level** | **<50 µM** | **50 to <100 µM** | **≥100 µM** | **No tHcy level** | **p values^a,b^** |  |
| **Total** | 633 | 601 | 278 | 212 | 111 | 32 |  | |
| **Thrombotic/thromboembolic, No. (%)** |  |  |  |  |  |  |  | |
| Any | 203 (32.1) | 186 (30.9) | 62 (22.3) | 85 (40.1) | 39 (35.1) | 17 (53.1) | <0.001 | |
| Cerebrovascular thromboembolic events (stroke/TIA) | 98 (48.3) | 87 (46.8) | 28 (45.2) | 42 (49.4) | 17 (43.6) | 11 (64.7) | 0.80 | |
| Deep vein thrombosis | 60 (29.6) | 58 (31.2) | 17 (27.4) | 25 (29.4) | 16 (41.0) | <5 | 0.32 | |
| Pulmonary embolism | 51 (25.1) | 46 (24.7) | 18 (29.0) | 20 (23.5) | 8 (20.5) | 5 (29.4) | 0.63 | |
| Arterial thrombosis | 17 (8.4) | 17 (9.1) | <5 | 11 (12.9) | 5 (12.8) | 0 (0.0) | 0.02 | |
| Myocardial infarction | 13 (6.4) | 13 (7.0) | 5 (8.1) | 7 (8.2) | <5 | 0 (0.0) | 0.53 | |
| Portal vein thrombosis | <5 | <5 | <5 | <5 | <5 | 0 (0.0) | 0.79 | |
| Sinus thrombosis | <5 | <5 | 0 (0.0) | <5 | <5 | 0 (0.0) | 0.49 | |
| Cerebral venous thrombosis | <5 | <5 | 0 (0.0) | <5 | 0 (0.0) | 0 (0.0) | >0.99 | |
| Renal vein thrombosis | <5 | <5 | 0 (0.0) | <5 | 0 (0.0) | 0 (0.0) | >0.99 | |
| **Skeletal, No. (%)** |  |  |  |  |  |  |  | |
| Any | 107 (16.9) | 100 (16.6) | 41 (14.7) | 38 (17.9) | 21 (18.9) | 7 (21.9) | 0.50 | |
| Fractures | 67 (62.6) | 66 (66.0) | 27 (65.9) | 25 (65.8) | 14 (66.7) | <5 | >0.99 | |
| Rib(s), sternum, thoracic spine | 17 (15.9) | 17 (17.0) | 8 (19.5) | 7 (18.4) | <5 | 0 (0.0) | 0.70 | |
| Wrist/hand level | 15 (14.0) | 15 (15.0) | 7 (17.1) | 6 (15.8) | <5 | 0 (0.0) | 0.82 | |
| Lower leg/ankle | 14 (13.1) | 14 (14.0) | <5 | <5 | 8 (38.1) | 0 (0.0) | 0.004 | |
| Foot/toe | 14 (13.1) | 14 (14.0) | 6 (14.6) | 6 (15.8) | <5 | 0 (0.0) | 0.87 | |
| Forearm | 9 (8.4) | 9 (9.0) | 5 (12.2) | <5 | 0 (0.0) | 0 (0.0) | 0.31 | |
| Skull/facial bones | 8 (7.5) | 8 (8.0) | <5 | <5 | <5 | 0 (0.0) | >0.99 | |
| Femur | <5 | <5 | 0 (0.0) | <5 | <5 | <5 | 0.32 | |
| Lumbar spine/pelvis | <5 | <5 | <5 | 0 (0.0) | <5 | 0 (0.0) | 0.15 | |
| Shoulder/upper arm | <5 | <5 | <5 | <5 | 0 (0.0) | 0 (0.0) | 0.43 | |
| Cervical vertebra/other parts of neck | <5 | <5 | <5 | 0 (0.0) | 0 (0.0) | 0 (0.0) | >0.99 | |
| Bone mass deficiency | 20 (18.7) | 18 (18.0) | 10 (24.4) | 5 (13.2) | <5 | <5 | 0.44 | |
| Osteoporosis | 20 (18.7) | 20 (20.0) | 9 (22.0) | 7 (18.4) | <5 | 0 (0.0) | 0.95 | |
| Scoliosis | 9 (8.4) | 8 (8.0) | <5 | <5 | <5 | <5 | 0.17 | |
| Pectus excavatum | 5 (4.7) | <5 | 0 (0.0) | 0 (0.0) | <5 | <5 | 0.21 | |
| Marfanoid habitus | <5 | <5 | 0 (0.0) | <5 | <5 | <5 | 0.06 | |
| Pes cavus | <5 | <5 | <5 | 0 (0.0) | 0 (0.0) | 0 (0.0) | >0.99 | |
| Genu valgum | 0 (0.0) | 0 (0.0) | 0 (0.0) | 0 (0.0) | 0 (0.0) | 0 (0.0) | N/A | |
| Kyphosis | 0 (0.0) | 0 (0.0) | 0 (0.0) | 0 (0.0) | 0 (0.0) | 0 (0.0) | N/A | |
| Pectus carinatum | 0 (0.0) | 0 (0.0) | 0 (0.0) | 0 (0.0) | 0 (0.0) | 0 (0.0) | N/A | |
| Skeletal fragility | 0 (0.0) | 0 (0.0) | 0 (0.0) | 0 (0.0) | 0 (0.0) | 0 (0.0) | N/A | |
| **Ocular, No. (%)** |  |  |  |  |  |  |  | |
| Any | 70 (11.1) | 63 (10.5) | 27 (9.7) | 24 (11.3) | 12 (10.8) | 7 (21.9) | 0.82 | |
| Glaucoma | 32 (45.7) | 26 (41.3) | 10 (37.0) | 11 (45.8) | 5 (41.7) | 6 (85.7) | 0.85 | |
| Myopia | 20 (28.6) | 19 (30.2) | 7 (25.9) | 7 (29.2) | 5 (41.7) | <5 | 0.64 | |
| Lens dislocation | 15 (21.4) | 13 (20.6) | 5 (18.5) | <5 | 6 (50.0) | <5 | 0.02 | |
| Cataracts | 12 (17.1) | 11 (17.5) | 5 (18.5) | 5 (20.8) | <5 | <5 | 0.83 | |
| Retinal detachment | 6 (8.6) | 6 (9.5) | 5 (18.5) | 0 (0.0) | <5 | 0 (0.0) | 0.06 | |
| Strabismus | <5 | <5 | <5 | <5 | 0 (0.0) | 0 (0.0) | 0.59 | |
| Iridodonesis | 0 (0.0) | 0 (0.0) | 0 (0.0) | 0 (0.0) | 0 (0.0) | 0 (0.0) | N/A | |
| **Neurological, No. (%)** |  |  |  |  |  |  |  | |
| Any | 56 (8.8) | 50 (8.3) | 16 (5.8) | 20 (9.4) | 14 (12.6) | 6 (18.8) | 0.06 | |
| Epilepsy | 37 (66.1) | 31 (62.0) | 7 (43.8) | 13 (65.0) | 11 (78.6) | 6 (100.0) | 0.16 | |
| Hemiplegia and hemiparesis | 12 (21.4) | 12 (24.0) | <5 | 6 (30.0) | <5 | 0 (0.0) | 0.64 | |
| White matter disease or lesions | 5 (8.9) | 5 (10.0) | <5 | 0 (0.0) | <5 | 0 (0.0) | 0.04 | |
| Aphasia | <5 | <5 | <5 | <5 | 0 (0.0) | 0 (0.0) | 0.54 | |
| Extrapyramidal and movement disorder | <5 | <5 | 0 (0.0) | 0 (0.0) | <5 | 0 (0.0) | 0.28 | |

Abbreviations: N/A, not applicable; tHcy, total homocysteine; TIA, transient ischemic attack.

^a^Comparison was assessed using linear regression with a generalized estimating equation and the Jonckheere-Terpstra test for continuous outcomes (means and medians, respectively), and Fisher’s exact test for categorical variables if a cell has a 0 value or frequency is ≤5, or Chi-square test.

^b^The no tHcy level subgroup was not included in the statistical test comparison.

The Optum minimum sample requirement is 5 or more subjects or patients for publications. Per guidance provided by Optum, all results indicated <5 are not shown due to small cell sizes.
